# Two Cases of Transcutaneous Electrical Nerve Stimulation of the Common Peroneal Nerve Successfully Treating Refractory, Multifactorial Leg Edema

**DOI:** 10.1177/2324709614559839

**Published:** 2014-11-20

**Authors:** Matthew V. Ingves, Adam H. Power

**Affiliations:** 1 Western University, London, ON, Canada

**Keywords:** edema, venous insufficiency, ulcer, electrical stimulation, refractory

## Abstract

The treatment of leg edema often involves promoting venous blood flow but can be difficult in patients with comorbidities that prevent traditional management strategies such as limb elevation or mechanical compression devices. The geko device is a self-contained neuromuscular stimulation device that adheres to skin over the common peroneal nerve and delivers a low-voltage stimulus that activates the lower-leg musculature resulting in enhanced superficial femoral vein blood flow and velocity. Here we report 2 cases of multifactorial and refractory leg edema successfully treated with the geko device over a period of 4 to 16 weeks. The device also improved pain and chronic wound healing. Although the geko device is costly, it was well tolerated and may provide another treatment strategy for resistant leg swelling.

## Introduction

Regardless of the etiology, enhancing venous blood flow is one of the strategies in treating leg edema. This can be accomplished by limb elevation,^1^ activating the calf muscle pump with exercise involving ankle plantar flexion,^2,3^ and mechanical devices such as compression garments^4-6^ or intermittent pneumatic compression devices.^7-9^ Unfortunately, these strategies may not be effective in some patients with comorbid conditions that limit limb movement or in patients with contraindications to adequate compression, such as skin infections and peripheral arterial insufficiency. In addition, current mechanical compression devices can be difficult to apply and uncomfortable, which decreases compliance.

An additional method to increase venous blood flow involves transcutaneous direct muscular stimulation. This uses electrical stimulation via electrodes applied to the skin to stimulate the calf muscle pump and promote venous blood flow,^10-12^ although results vary when compared with voluntary ankle contraction.^13,14^ Because of the high voltage intensity required and subsequent discomfort, these devices have not been widely adopted in routine clinical use. The geko device (FirstKind Ltd, High Wycombe, United Kingdom) is an alternative to direct electrical muscle stimulation for activating the calf muscle pump.^15^ It is a self-contained neuromuscular stimulation device that adheres to skin over the common peroneal nerve and delivers a low-voltage stimulus at a frequency of 1 Hz, thereby activating the calf and foot musculature of the lower limb without the voltage-related discomfort. The geko device significantly increases superficial femoral vein blood flow and velocity^15^ and provides a possible safe and tolerable method to treat leg edema.

Here we report successful treatment with the geko device in 2 patients with refractory, multifactorial leg edema. This report was ethically approved by Western University’s Research Ethics Board for Health Sciences Research Involving Human Subjects, and informed patient consent was obtained while keeping patient identifying information anonymous.

## Case 1

**Figure 1.**
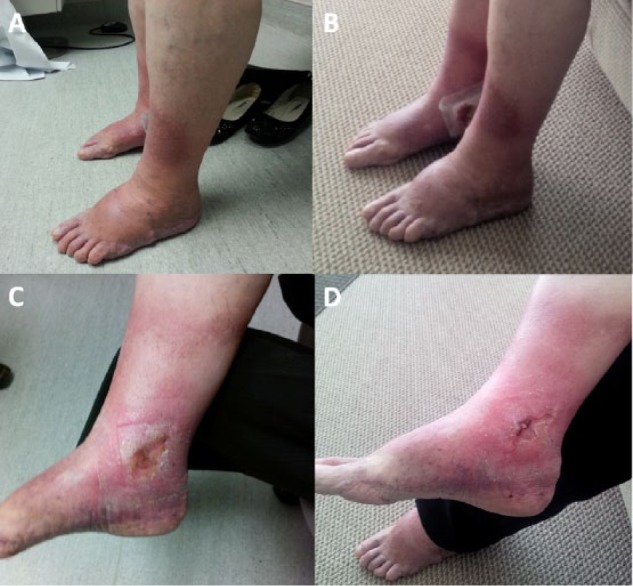
Photographs displaying multifactorial bilateral lower-extremity edema and swelling before (A) and following (B) 10 weeks of bilateral transcutaneous electrical nerve stimulation treatment with the geko device in a 60-year-old woman; (C) and (D) illustrate healing of the patient’s chronic ulcer before and after the 10-week course of geko therapy, respectively. The device was placed over the common peroneal nerve and resulted in a reduction in leg swelling and symptomatology.

Our first patient was a 60-year-old woman with multiple venous and arterial risk factors, including remote bilateral lower-leg deep venous thromboses, varicose veins, lymphedema, and coronary artery disease with 2 myocardial infarctions. She was on furosemide for her heart disease and congestive heart failure. She presented with bilateral disabling lower-extremity edema and a right ankle ulcer for >1 year. Additionally, there was cellulitis present on the right foot, extending to above the ankle. She had multiple varicosities and skin changes consistent with chronic venous insufficiency but also features in keeping with chronic lymphedema in both feet. Her arterial circulation demonstrated weakly palpable lower-extremity pulses and ankle-brachial indices were only mildly reduced. Duplex ultrasound showed deep venous reflux but no superficial venous insufficiency. Compression wrappings, exercise, and leg elevation did not effectively improve her pain or ulceration over the past year. Because of a failure of conservative traditional management, a 10-week trial with the geko device applied bilaterally over the common peroneal nerve was initiated. The device produced muscle twitches of the tibialis, peroneus longus, and gastrocnemius muscles. The patient initially wore the geko device for 2 hours a day, but within 6 weeks, she had progressed to wearing the device 4 hours a day. She reported a positive time-dependent response to geko therapy. At the 10-week follow-up assessment, there was a reduction in leg swelling that was accompanied by relief of symptoms and improved right ankle ulcer healing (Figure 1). Her calf diameters, which initially measured 43 cm on the left and 45 cm on the right, decreased to 41 cm (5%) and 40 cm (13%), respectively. Her ankles, which initially measured 31 cm on the left and 32 cm on the right, both decreased to 29 cm (7%) and 30 cm (7%), respectively. Her ulcer dimensions decreased from 4.1 × 4.7 cm^2^ to 3.3 × 4.0 cm^2^. Continued use of the geko device resulted in almost complete resolution of her swelling, and after 16 weeks, she was able to resume an exercise and leg elevation regimen for maintenance without the geko device.

## Case 2

**Figure 2.**
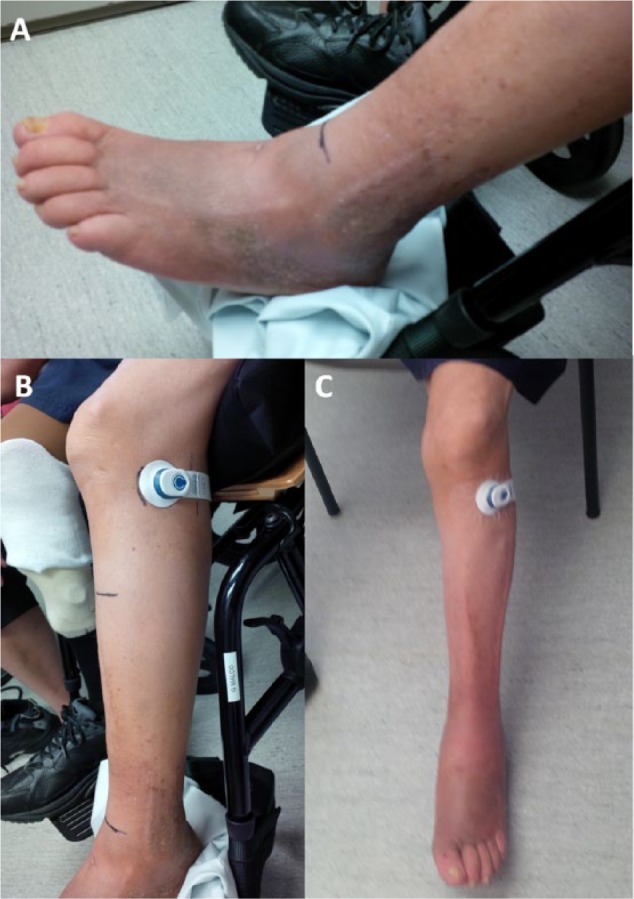
Photographs before (A and B) and following (C) a 4-week course of transcutaneous electrical nerve stimulation using the geko device to treat left leg below-knee swelling and pain in a 70-year-old man. Placement of the device over the common peroneal nerve (B and C) activated the short head of the biceps femoris muscle.

The second patient was a 70-year-old man with complex left leg swelling and pain. He had a Charcot left foot deformity, foot ulcers, and peripheral neuropathy secondary to diabetes mellitus. He wore a leg brace and an orthotic boot on his left leg. A right diabetic foot infection led to a right below-knee amputation. He had multiple reasons for lower-extremity edema, including a significant cardiac history, renal failure, chronic venous insufficiency, and liver cirrhosis. The patient presented with a 1-month history of left lower leg edema below the knee. Consequently, he was no longer able to fit into his left leg brace and orthotic boot, and he developed significant pain that limited his ability to ambulate and elevate his leg for prolonged periods. The swelling in his left lower leg was maximal in the ankle and foot (Figure 2). Because of failure of traditional management, we proceeded with a trial of the geko device to treat his leg swelling.

Initial placement of the geko device over its intended site did not stimulate the calf muscles. We suspected that this was a consequence of diabetic peripheral neuropathy of his common peroneal nerve. Instead, the geko device stimulated the short head of the biceps femoris muscle. Following 4 weeks of daily geko use, there was almost complete resolution of the left lower leg swelling, with the majority of improvement occurring during the fourth week of therapy (Figure 2). During this time, the device produced minimal discomfort for the patient. The initial left calf circumference decreased from 29 to 23 cm (21% reduction). The patient was ambulating, had significant improvement in leg pain, and was able to fit into his orthotic boot. Given the improvement in leg swelling and pain, the geko device was discontinued, and the patient was instructed to wear his orthotic boot when ambulatory and to resume leg elevation and exercise.

## Discussion

These case reports demonstrate the successful treatment of multifactorial, refractory lower-leg swelling using transcutaneous electrical nerve stimulation over the common peroneal nerve with differing muscle activation. Each patient had unique medical circumstances that contributed to failure of traditional edema management, but both responded to daily treatment using a novel, self-contained, and lightweight transcutaneous electrical nerve stimulation device. Results were evident within 4 weeks of treatment initiation, and the device was well tolerated, with the patients noticing minimal pain and discomfort.

Historically, transcutaneous electrical nerve stimulation has been used for the treatment of pain, which targets sensory nerves.^16^ In contrast, transcutaneous electrical muscular stimulation targets the musculature of the lower extremities directly (eg, gastrocnemius muscle) with the goal of promoting venous blood flow to treat leg edema and prevent venous thrombosis.^10,12,17-19^ Direct muscular electrical stimulation increases venous blood flow^20,21^ but requires higher voltage stimuli and is difficult for patients to tolerate.^22^ Moreover, direct muscle stimulation is less effective at evoking a response because only one muscle group is targeted. Conversely, recruiting multiple muscle groups in the lower extremity by targeting motor nerves may be more beneficial.^15,22,23^ Data from healthy individuals demonstrate that transcutaneous electrical nerve stimulation via the common peroneal nerve significantly enhances venous flow, although there was no advantage shown over other conventional mechanical methods of calf stimulation.^22^ Because patients tolerate transcutaneous electrical nerve stimulation more than direct muscle stimulation, improved compliance is expected. Our patients tolerated the geko device well, with no adverse effects reported.

The geko device offers many advantages over other stimulation devices: it is compact, lightweight, and portable because it requires no external power source or cords. Moreover, it has a 24-hour battery life and is easy to apply and use. Because a new device is required daily, the geko device can, however, be costly. Its tolerability and potential clinical utility has been evaluated in cohorts of healthy volunteers,^15,23^ including those wearing below-knee plaster casts, indicating the potential for use in conditions with limited space where other, more bulky devices, such as intermittent pneumatic compression devices, may not be suitable.

Our cases are unique in that most previous experimental findings were reported in young healthy volunteers. Our patients had many comorbid conditions, indicating that there is a potentially diverse patient population that the geko device might be beneficial for. We have shown that the device effectively and efficiently treated lower-leg swelling in patients with multifactorial leg edema when traditional treatments failed. This device can be considered in patients with refractory leg edema.
